# Injection-Molded Carbon Fiber/Carbon Nanotube-Reinforced Polypropylene Composite Electrodes: Processing–Structure–Property Relationships and Aqueous Electrochemical Applications

**DOI:** 10.3390/polym18182311

**Published:** 2026-09-21

**Authors:** Minseok Kim, Dongwon Lee, Wonhyun Kwon, Joon Young Kim, Dongwon Kim, Daewoong Seo, Hyungwook Park, Yeon Uk Jeong, Jong Won Shin

**Affiliations:** 1 Korea Institute of Science and Technology Information (KISTI), Daejeon 34141, Republic of Korea; 2 Changlim ENG Co., Ltd., Daegu 42703, Republic of Korea; 3 Climate AI Energy Laboratory, Jeonnam Research Institute, Naju 58245, Republic of Korea; 4 Beamline Department, Pohang Accelerator Laboratory, POSTECH, Pohang 37673, Republic of Korea; 5 School of Materials Science and Engineering, Kyungpook National University, Daegu 41566, Republic of Korea

**Keywords:** conductive polymer composite, polypropylene, carbon fiber, carbon nanotube, injection molding, water electrolysis, electrochemical disinfection, *Escherichia coli*

## Abstract

Conductive polymer composites offer a scalable route to self-supporting electrochemical electrodes, but electrical performance must be balanced against processability at high filler loadings. Fifteen polypropylene (PP)-based carbon-filler formulations were melt-compounded and injection-molded, and their electrical resistivity and moldability were evaluated. PP/CF20/CNT20 was selected, exhibiting a mean volume resistivity of 0.1409 Ω·cm with complete mold filling, no visible surface defects, and good moldability. SEM and synchrotron X-ray CT revealed multiscale carbon-filler morphology and spatially heterogeneous CF organization. Perforated PP/CF20/CNT20 disks were assembled into a ten-electrode parallel stack. At 12 V, the voltage sweep reached approximately 7.1 A in 1 M KOH and 2.1 A in tap water; during 20 h fixed-voltage operation, currents stabilized at approximately 5.2–5.5 A and 2.1 A, respectively. In 1 M KOH, estimated H_2_ flow reached approximately 42.9 sccm at 7 A. Prolonged tap-water electrolysis produced Ca-dominated surface scale. At 12 V in 0.3 wt.% NaCl, no *Escherichia coli* colonies were detected under the applied plating conditions from 15 min onward. These results demonstrate the feasibility of injection-molded PP/CF/CNT composites as functional electrodes for aqueous electrochemical applications.

## 1. Introduction

Conductive polymer composites (CPCs) combine the low density, chemical resistance, and scalable processability of thermoplastics with the electrical functionality of carbon fillers. In melt-processed CPCs, charge transport arises when carbon fibers (CFs), carbon nanotubes (CNTs), graphite (Gr), or carbon black (CB) establish continuous or tunneling-assisted conductive networks. In PP-based conductive composites, both filler composition and processing conditions strongly influence electrical and mechanical performance. Tariq et al. [1] demonstrated that hybrid carbon-filler formulations can balance electrical conductivity and flexural strength in PP-based bipolar plates, while Chen et al. [2] showed that injection-molding conditions affect the electrical and mechanical properties of CF/PP composites.

Polypropylene (PP) is an excellent matrix candidate because of its low density, low moisture uptake, chemical resistance, recyclability, and compatibility with melt compounding and high-throughput injection molding. The properties of molded PP composites are further affected by filler morphology and orientation; CNT orientation can produce anisotropic electrical behavior, whereas CF reinforcement can substantially improve mechanical stiffness [3,4].

Carbon fillers provide complementary functions because of their distinct dimensions and morphologies. Filler type and hybrid-filler combinations strongly influence electrical percolation and resistivity in PP composites [5,6], while CNT incorporation into CF/PP systems can also modify interfacial and mechanical properties [7].

The processing route is critical for translating these filler functions into a usable molded component. Premixing and melt compounding influence filler wetting, dispersion, and fiber attrition, whereas injection molding imposes additional shear and extensional flow that can orient CFs and redistribute CNT-containing domains. High-shear molding can further alter conductive-phase morphology and electrical conductivity in PP-based composites [8]. Increasing filler loading can improve conductive-network formation but can also increase melt viscosity, promote agglomeration, and impair complete mold filling [1,3]. Formulation selection should therefore consider both the electrical performance and injection moldability of the final molded component rather than filler content or conductivity measured only in an intermediate material.

Conductive thermoplastic/carbon composites have been developed for bipolar plates and other current-carrying components [1]. More recent studies have extended this concept to continuously processed conductive electrodes and films, further demonstrating the influence of processing route and hybrid filler design on final electrical performance [9,10]. Table 1 summarizes representative molded and extruded systems [10,11,12].

Because these literature values were obtained using different specimen forms and test configurations, Table 1 is intended to illustrate the reported range and common reporting practices rather than to provide a direct performance ranking. This variability highlights the importance of evaluating electrical functionality in the final processed component, particularly when the component is intended to function as an electrode rather than solely as a passive conductor.

Polymer–carbon composites have also been investigated as active electrodes in aqueous electrochemical systems. Liu et al. prepared polyethylene/graphite composite electrodes and demonstrated their operation as both anodes and cathodes in electroosmotic dehydration and electro-Fenton processes; electrode lifetime depended on current density, agitation, electrode thickness, and gas-bubble accumulation [13]. More recently, Volz et al. developed free-standing conductive polymer-composite catalyst layers containing a matrix polymer, carbon filler, and nickel catalyst for anion-exchange-membrane water electrolysis, achieving operation at current densities above 3 A cm^−2^ [14]. These studies demonstrate that polymer-based conductive composites can sustain electrochemical reactions in aqueous environments. However, these systems differ from self-supporting bulk PP/carbon composites produced by melt compounding and injection molding, for which direct electrolysis performance remains less well documented.

**Figure 1:**
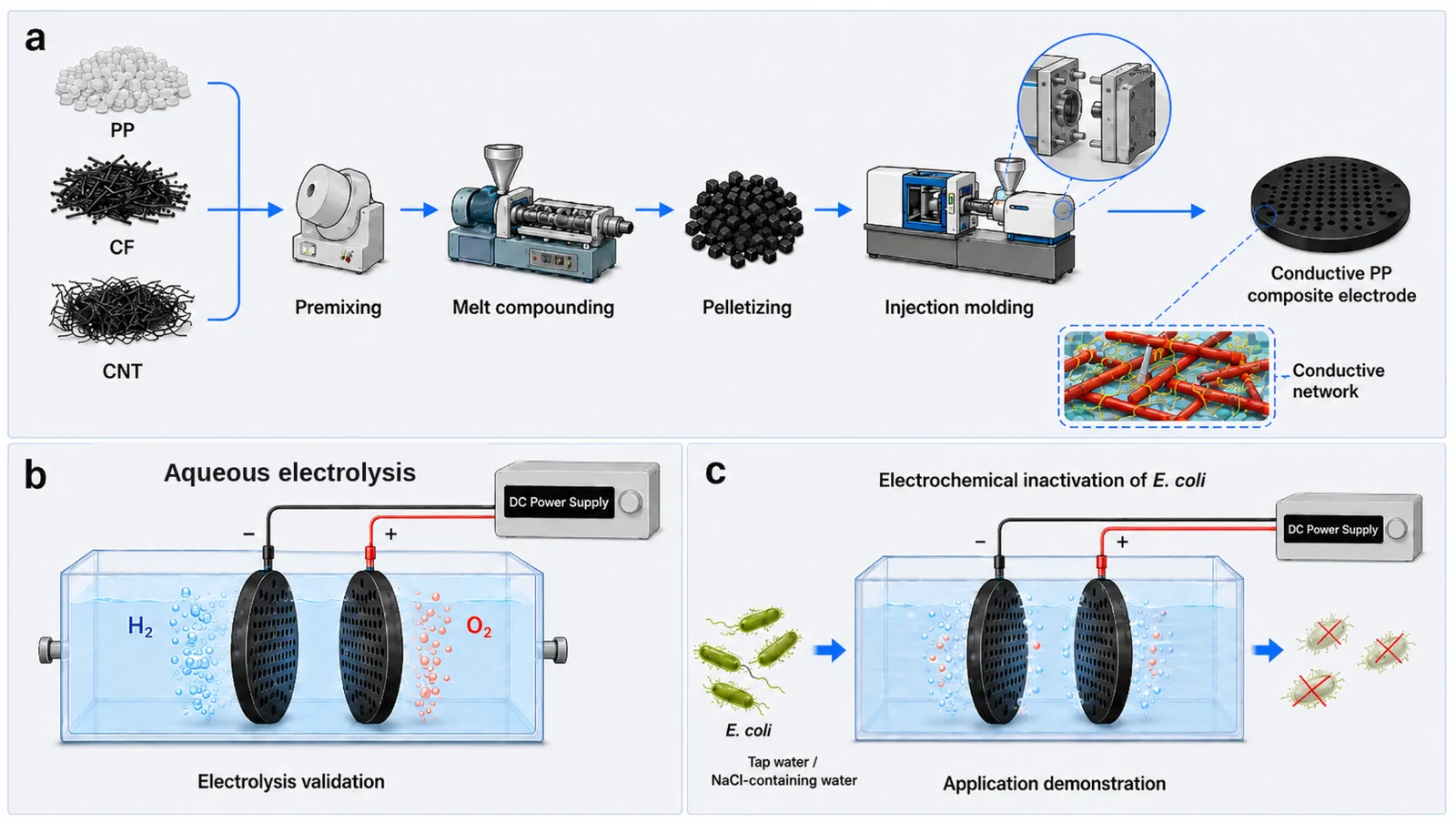
Schematic overview of the electrode fabrication and application pathway: (**a**) PP/carbon-filler premixing, melt compounding, pelletization, and injection molding; (**b**) assembly and aqueous electrolysis testing of the perforated composite electrodes; and (**c**) exploratory evaluation of *Escherichia coli* inactivation.

Accordingly, the primary objective of this study was to develop an electrically conductive PP-based composite that could be melt-compounded and injection-molded into a functional electrochemical electrode. To achieve this, we selected PP as the matrix due to its robust processability and chemical resistance and employed carbon fillers—specifically CF and CNT—to exploit their complementary electrical and structural characteristics. Fifteen PP/carbon-filler formulations were screened on the basis of the volume resistivity and injection-molding behavior of the final molded specimens, and a CF/CNT formulation was selected for detailed electrical, mechanical, and microstructural characterization. The selected composite was subsequently injection-molded into perforated electrode disks and evaluated under aqueous electrolysis conditions. Current–voltage response, sustained current operation, and gas evolution in tap water and 1 M KOH were used as the primary functional validation of aqueous electrolysis [14,15]. Operation in tap water and salt-containing media was considered separately because dissolved ionic species can alter solution conductivity and participate in additional electrode reactions or precipitation processes [16,17,18]. Finally, electrochemical inactivation of *Escherichia coli* was included as an application-level demonstration of potential water-treatment utility rather than as a comprehensive mechanistic disinfection study [16,19,20,21,22,23,24,25]. The overall fabrication and application pathway is illustrated in Figure 1.

## 2. Materials and Methods

### 2.1. Materials and Formulation Design

Polypropylene (PP; HOPELEN J-170H, LOTTE Chemical Corporation, Seoul, Republic of Korea) was used as the thermoplastic matrix. J-170H is an injection-molding-grade PP homopolymer with a nominal melt flow index of 30 g/10 min (ASTM D1238) and a density of 0.90 g/cm^3^ (ASTM D792).

Chopped carbon fiber (CF; ACECA-CBZ PU, ACE C&TECH Co., Ltd., Yeongju, Republic of Korea) was used as the reinforcing and conductive fibrous filler. The CF was produced from Zoltek 50K carbon-fiber yarn and had a nominal chopped length of 6 ± 2 mm. The fibers were supplied with a polyurethane sizing; the certificate of analysis for the material used in this study reported a sizing content of 5.24 wt.% and a bulk density of 0.52 g/cm^3^.

Multiwalled carbon nanotubes (CNTs; K-Nanos 100T, Kumho Petrochemical Co., Ltd., Seoul, Republic of Korea) were used as the nanoscale conductive filler. The CNTs had a nominal diameter of 8–15 nm, a bundle diameter of 1–15 µm, a bundle length of 10–50 µm, an average purity of approximately 95%, and a bulk density of 0.06–0.14 g/mL.

Crystalline graphite (Gr; SHC-99325, Seongha Yeongdong Plant, Yeongdong, Republic of Korea) and carbon black (CB; Chezacarb AC-60, UNIPETROL RPA, s.r.o., Litvínov, Czech Republic) were also used in selected formulations during the initial screening stage. The graphite had a free-carbon content of 99% and a nominal size specification of approximately 325 mesh. Chezacarb AC-60 had a nitrogen-adsorption specific surface area of at least 800 m^2^/g and a DBP absorption of at least 380 mL/100 g.

All formulations were defined as weight percentages of the total compounding feed, with PP constituting the balance. Fifteen PP-based formulations containing single or hybrid carbon fillers were prepared to evaluate their suitability for injection molding and electrical conduction.

### 2.2. Premixing, Melt Compounding, and Pelletization

To reduce segregation between fine carbon fillers and PP pellets, the matrix resin was mechanically or cryogenically ground before mixing. The resin and fillers were weighed according to the designated formulations and premixed in a 100 L high-speed mixer at 500 rpm for 5 min, using a minimum batch size of 10 kg. Mixtures containing powdered fillers, including CNTs and graphite, were dispersed in methanol by ultrasonication in an ultrasonic bath (DWH-20K, Daewon Ultrasonic, Siheung, Republic of Korea) at 20 kHz and 1000 W for 1 h before subsequent homogenization in a continuous mixer.

The premixed materials were melt-compounded using a Buss-type SJW45 single-screw co-kneader/extruder (XINDA, Jiangsu Xinda Tech Limited, Wuxi, China). To limit CF breakage during compounding, PP and powdered fillers were supplied through the main hopper, whereas CF was introduced separately through the side feeder. For CF-only formulations, PP was supplied through the main hopper and CF through the side feeder. For hybrid formulations, PP and the powdered fillers were premixed and supplied through the main hopper, whereas CF was introduced through the side feeder. Representative SJW45 processing temperatures were 160, 230, 235, 240, and 240 °C for zones 1–5, respectively, with a die-head temperature of 240 °C. The melt-pump and main-motor frequency settings were 4.5 and 10 Hz, respectively, and the material-feed setting was 5.5–6.0 Hz. The extruded strands were air-cooled with a blower and subsequently pelletized at 310–350 rpm.

Following pelletization, the compounded PP materials were oven-dried at 90–140 °C for 10 h, depending on the formulation. Moisture content was measured using a moisture analyzer, and only pellets with a moisture content below 0.02% were used for injection molding.

### 2.3. Injection Molding and Electrical-Resistivity Screening

Compounded pellets from the fifteen formulations were injection-molded under formulation-specific processing conditions. Barrel and nozzle temperatures of 210–300 °C, injection pressures of 58–80 bar, and holding pressures of 35–80 bar were used across the fifteen formulations. Injection-molding outcomes were recorded qualitatively in terms of mold filling, visible surface defects, and overall moldability. For PP/CF20/CNT20, the barrel-zone temperatures were 230/240/240/240 °C, the injection-speed setting was 50%, the injection pressure was 80 bar, and the holding pressure was 60 bar.

Electrical resistance was measured using a four-point-probe system (CMT-SR3000S, AIT, Suwon, Republic of Korea). The probe head had a pin spacing of 1 mm, a pin load of 100 g, and a pin-tip radius of 100–150 µm. The injection-molded specimens had a nominal thickness of 3.175 mm, and volume resistivity (Ω·cm) was determined from the specimen thickness using the instrument’s built-in resistivity-calculation function. Because the four collinear probe tips contacted the same molded surface, the measurement represents a surface/in-plane electrical response rather than a through-plane measurement.

For each formulation, five separate injection-molded specimens were evaluated. Five repeated measurements were obtained at distributed positions on the molded surface of each specimen, yielding 25 readings per formulation. The five readings from each specimen were averaged to obtain a specimen-level value, and the mean ± SD of the five specimen-level values was reported in Table 2 (*n* = 5 specimens). Formulations were screened on the basis of mean volume resistivity together with successful injection molding. Electrical conductivity was calculated, where required, as the reciprocal of volume resistivity:

*σ* = 1/*ρ* (1)

where σ is the electrical conductivity and ρ is the volume resistivity.

### 2.4. Mechanical Characterization

The PP/CF20/CNT20 composite was subjected to mechanical characterization after injection molding. Tensile strength and elongation were determined according to ASTM D638, and flexural strength and flexural modulus according to ASTM D790. The values reported in Table 3 are representative values from the mechanical-characterization dataset.

### 2.5. Microstructural Characterization

The microstructure of the injection-molded PP/CF20/CNT20 composite was examined by scanning electron microscopy (SEM) at the Korea Basic Science Institute (KBSI), Daegu Center, Republic of Korea, using a Scios DualBeam focused ion beam/scanning electron microscope (FEI, Hillsboro, OR, USA) operated in SEM mode. Fracture surfaces were examined at an accelerating voltage of 5.0 kV and a probe current of 0.10 nA using an Everhart–Thornley detector. The working distance was approximately 9.3–9.9 mm, and images were acquired at magnifications up to 20,000×.

The SEM images were evaluated qualitatively for CF distribution and orientation, fiber pull-out, matrix morphology, fiber–matrix interfaces, fibrillar features, and voids. Torn or sheet-like regions surrounding the fibers were interpreted as PP-rich matrix regions.

Synchrotron X-ray computed tomography (CT) was performed at the 9D white X-ray beamline of the Pohang Light Source-II (PLS-II), Pohang Accelerator Laboratory (PAL), Republic of Korea, to examine the three-dimensional internal structure of the composite. The 9D beamline uses bending-magnet white synchrotron X-rays with an approximate photon-energy range of 4–50 keV [26]; PLS-II operates at an electron energy of 3 GeV and a stored beam current of 400 mA. CT data were acquired with an exposure time of 50 ms over 901 projections using a 4 mm DSP-wafer attenuator and a sample-to-detector distance of 20 cm. The reconstructed coordinate system and representative center and edge observation regions were defined for the CT assessment. For each region, reconstructed three-dimensional volumes were inspected from two representative viewing angles to assess the bulk-scale spatial organization of CF-scale features. The CT assessment was qualitative: individual fibers were not segmented, and fiber-orientation tensors, local fiber volume fractions, and through-thickness orientation profiles were not calculated. SEM and CT specimens were obtained from the final PP/CF20/CNT20 compounding and injection-molding batch used for the electrical and mechanical-property evaluations.

### 2.6. Electrode Fabrication and Electrolysis Cell

A dedicated injection mold was designed and fabricated for repeated production of the composite electrode disks. PP/CF20/CNT20 pellets were injection-molded into circular perforated disks with a diameter of 100 mm and a thickness of 3.0 mm. Each disk contained 220 through-holes with a diameter of 2 mm to increase the electrolyte-accessible geometric area and facilitate gas release.

**Figure 2:**
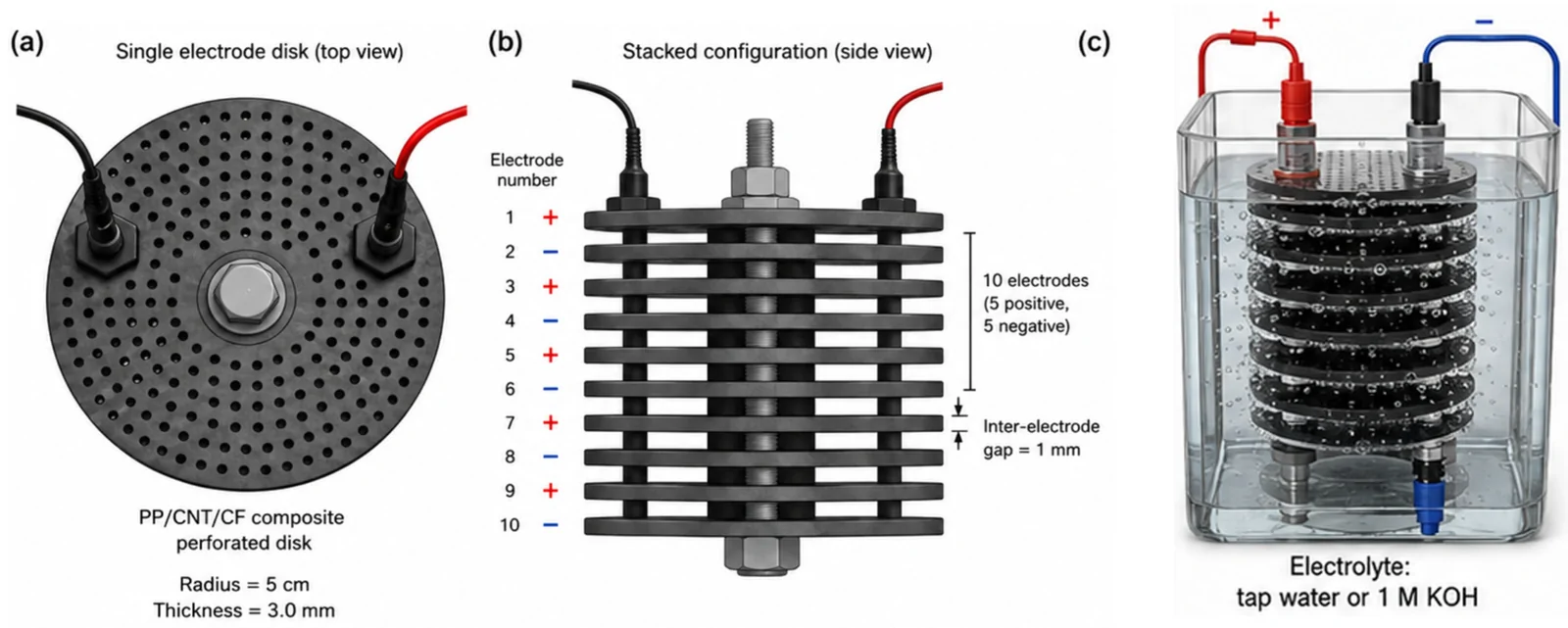
Configuration of the parallel-connected PP/CF20/CNT20 electrode stack: (**a**) injection-molded perforated disk; (**b**) alternating stack of five positively and five negatively connected electrodes; and (**c**) assembled stack immersed in tap water or 1 M KOH.

Identical composite disks were used as both anodes and cathodes, with polarity defined by the external electrical connection. Ten electrodes, comprising five positively connected and five negatively connected disks, were arranged alternately in a vertical stack with a fixed interelectrode spacing of 1 mm, giving nine electrolyte-filled gaps. All positive electrodes were connected to the positive terminal and all negative electrodes to the negative terminal, forming a parallel-connected multi-electrode assembly. The metallic electrical-connection hardware was maintained above the electrolyte level and did not come into direct contact with the electrolyte. This electrode geometry and ten-electrode parallel-stack architecture were used throughout the electrolysis and electrochemical-inactivation experiments described below. The configuration of the injection-molded electrode disk and the ten-electrode parallel stack is shown in Figure 2.

### 2.7. Current–Voltage and Gas-Evolution Characterization of the Parallel-Connected Stacked Electrode System

The current–voltage (I–V) characteristics of the injection-molded PP/CF20/CNT20 composite electrodes were evaluated using the ten-electrode parallel-connected stack described in Section 2.6.

The assembled electrode stack was fully immersed in either tap water or 1 M KOH aqueous electrolyte. A programmable DC power supply (9205B Series, B&K Precision Corporation, Yorba Linda, CA, USA; maximum output: 60 V, 25 A, and 600 W) was used to apply the cell voltage across the stacked electrode assembly. The power supply provides voltage and current programming/readback resolutions of 1 mV and 0.1 mA, respectively. The applied voltage was linearly swept from 0 to 12 V at a scan rate of 0.1 V s^−1^, while the corresponding total cell current was continuously recorded. The same electrode number, interelectrode spacing, stack geometry, electrical connection scheme, and voltage-sweep conditions were maintained for both electrolyte systems to enable direct comparison of their I–V responses.

For the long-duration current test, the same stack was operated at a fixed cell voltage of 12 V for 20 h in each electrolyte, and the total cell current was recorded continuously.

The gases evolved during electrolysis were collected without separation and passed through a gas flow meter (TSM-D210D, MK Precision, Siheung, Republic of Korea; nominal H_2_ flow capacity: 300 sccm). Because both hydrogen and oxygen generated at the cathodic and anodic electrodes, respectively, were introduced into the flow meter as a mixed gas stream, the measured flow rate represented the combined evolved gas flow rather than a directly measured hydrogen flow rate. Assuming that the collected gas consisted solely of H_2_ and O_2_ with the theoretical stoichiometric volume ratio of H_2_:O_2_ = 2:1, the hydrogen flow rate was estimated as two-thirds of the measured mixed-gas flow rate according to

Q_H2,est_ = 2/3 Q_mixed_ (2)

where Q_mixed_ is the measured mixed-gas flow rate and Q_H2,est_ is the estimated hydrogen flow rate. The gas flow rate was recorded simultaneously with the applied voltage and total cell current to correlate gas evolution with the electrical response of the stacked electrode system.

The measured voltage represents the potential difference applied across the entire parallel-connected electrode assembly rather than the potential of an individual electrode, whereas the measured current corresponds to the total current drawn by the complete stacked system. The estimated hydrogen flow rate, derived from the measured mixed-gas flow based on the theoretical H_2_:O_2_ volume ratio of 2:1, was used to evaluate the onset and evolution of hydrogen generation as a function of the applied cell voltage. The resulting I–V and gas-evolution characteristics were used to evaluate the cell-level electrical and electrochemical response of the PP/CF20/CNT20 stacked electrode system and to investigate the influence of electrolyte ionic conductivity on current transport and hydrogen evolution under otherwise identical operating conditions.

### 2.8. Experimental Procedure for Electrochemical Inactivation of Escherichia coli

The same ten-electrode parallel-connected PP/CF20/CNT20 stack described in Section 2.6 was used for the *E. coli* inactivation experiments. A fixed cell voltage of 12 V was applied throughout all *E. coli* inactivation experiments. *Escherichia coli* ATCC 15489 was cultured in nutrient broth at 37 °C for 48 h. The cells were harvested by centrifugation, washed, and used to prepare the bacterial inoculum. The *E. coli* tests were conducted in ion-containing water matrices under three conditions: (i) 10 L of tap water inoculated with 5 mL of the bacterial culture; (ii) 20 L of ultrapure water containing 0.1 wt.% NaCl and inoculated with 20 mL of culture; and (iii) 10 L of ultrapure water containing 0.3 wt.% NaCl and inoculated with 10 mL of culture. The tap water used in the experiments was potable municipal water; available water-quality information indicated a residual chlorine concentration of approximately 0.2 mg/L (0.2 ppm). Free and total chlorine concentrations were not measured concurrently during the microbiological treatment. Viable counts were evaluated at the common treatment times of 0, 15, 30, and 60 min.

At each sampling time, a 1 mL sample was collected and neutralized with a sodium thiosulfate/sodium thioglycolate solution. Viable bacteria were enumerated by the pour-plate method using nutrient agar, followed by incubation at 37 °C for 48 h, and were expressed as colony-forming units per milliliter (CFU/mL). Each water-matrix condition was evaluated in three independent electrochemical inactivation experiments, and five replicate plates were prepared for each sampled experiment. Because the initial viable counts and test volumes differed among conditions, treatment responses were evaluated relative to the initial viable count (N_0_) for each condition. For quantifiable observations, the log-reduction results from the three independent experiments were summarized as mean ± SD (*n* = 3). The dilution scheme used in the archived count records was not available; consequently, the quantitative detection limit could not be reconstructed. Samples for which no colonies were observed under the applied plating conditions are therefore reported as not detected (ND) rather than interpreted as complete bacterial elimination.

The percentage reduction in viable bacterial concentration at treatment time t was calculated relative to the initial concentration N_0_ as

Reduction (%) = [(*N*_0_ − *N*_t_)/*N*_0_] × 100 (3)

where N_t_ is the viable bacterial concentration at treatment time t. For the logarithmic presentation, log reduction was calculated for quantifiable counts as log_10_(N_0_/N_t_). ND observations were not assigned numerical log-reduction values because the quantitative detection limit was unavailable.

### 2.9. Post-Electrolysis Electrode and Deposit Characterization

**Figure 3:**
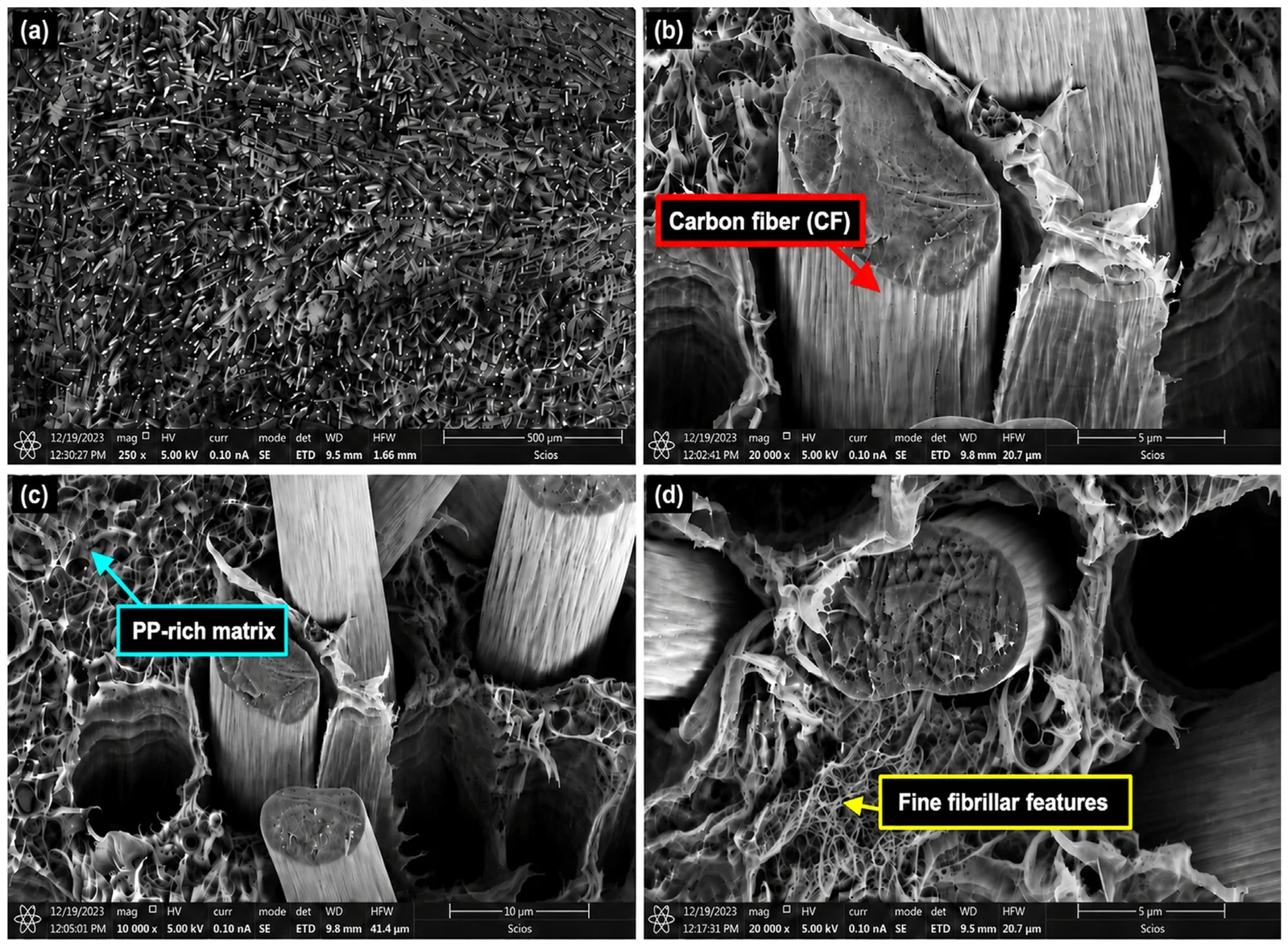
Representative SEM images of the fracture surface of injection-molded PP/CF20/CNT20: (**a**) low-magnification overview; (**b**) carbon fiber; (**c**) a PP-rich matrix region; and (**d**) fine fibrillar features near a PP/CF interface.

Photographs of unused and electrolysis-exposed PP/CF20/CNT20 electrodes obtained in this study were compared to visually assess surface-scale formation after 120 h of continuous operation in tap water at 12 V. Representative fracture-surface images of the unused composite are presented in Figure 3. For the used electrode, fracture-surface SEM images were acquired at nominal magnifications of 1000×, 5000×, and 50,000×; two independent fields were imaged at 1000×. Because the unused and used SEM observations were obtained from different physical locations, the comparison was restricted to qualitative morphological assessment.

A cathodic scale sample collected after electrolysis was analyzed by wavelength-dispersive X-ray fluorescence (WDXRF) using an S8 TIGER spectrometer (Bruker AXS, Karlsruhe, Germany) equipped with a 4 kW Rh-target X-ray source at the Korea Basic Science Institute (KBSI), Daegu Center, Republic of Korea. The XRF data were used to determine the dominant elemental composition on an oxide-equivalent basis. Because XRF alone does not identify crystalline phases, the deposit was interpreted in terms of elemental composition rather than assigned to a specific mineral phase.

## 3. Results and Discussion

### 3.1. Formulation Design and Electrical-Resistivity Screening of Injection-Molded Composites

The fifteen formulations were evaluated after the complete compounding–pelletization–injection-molding sequence to determine the electrical performance retained in the final molded specimens. Mean volume resistivity varied from 0.1261 to 2.2730 Ω·cm (Table 2), indicating a strong dependence on formulation.

The lowest mean resistivity was obtained for PP/CF30/CNT10/Gr10 (0.1261 Ω·cm), followed by PP/CF25/CNT10/Gr15 (0.1329 Ω·cm) and PP/CF20/CNT20 (0.1409 Ω·cm). Because the screening matrix was designed for practical formulation comparison rather than as a factorial experiment, these differences are interpreted as formulation-level performance rather than as quantitative evidence of individual filler synergy.

Formulation selection therefore considered electrical resistivity together with the qualitative injection-molding outcomes summarized in Table 2. Among the three lowest-resistivity formulations, PP/CF30/CNT10/Gr10 and PP/CF25/CNT10/Gr15 were rated as difficult to mold and showed visible surface defects, whereas PP/CF20/CNT20 achieved complete mold filling with no visible surface defects and was rated as having good overall moldability. This processing criterion is relevant for highly filled conductive PP composites because increasing carbon-filler loading can increase melt viscosity and impair mold filling [1,2,3,27].

### 3.2. Selection and Mechanical Properties of the Final Injection-Molded Composite

Although PP/CF20/CNT20 did not exhibit the lowest mean resistivity among the fifteen formulations, its mean volume resistivity of 0.1409 Ω·cm was only modestly higher than those of PP/CF30/CNT10/Gr10 (0.1261 Ω·cm) and PP/CF25/CNT10/Gr15 (0.1329 Ω·cm). Unlike these two lower-resistivity formulations, which were rated as difficult to mold and exhibited visible surface defects, PP/CF20/CNT20 showed complete mold filling, no visible surface defects, and good overall moldability. PP/CF20/CNT20 was therefore selected as the final formulation based on its combined electrical performance and injection-molding outcome rather than resistivity ranking alone.

The final injection-molded PP/CF20/CNT20 composite exhibited a tensile strength of 100.9 MPa, a tensile elongation of 1.0%, a flexural strength of 168.5 MPa, and a flexural modulus of 18.87 GPa (Table 3). The combination of high stiffness and low elongation is consistent with the reinforcement–ductility trade-off reported for carbon-filled PP systems. Stanciu et al. [27] reported that increasing MWCNT loading in injection-molded PP increased tensile modulus and strength while markedly reducing strain at break, and Arao et al. [7] likewise demonstrated mechanical reinforcement in injection-molded CF/PP composites hybridized with nanofillers.

For the present electrode design, the measured flexural strength and modulus are relevant to the dimensional rigidity of the large, perforated electrode because excessive deformation could alter the electrode geometry and the designed interelectrode spacing during handling and cell assembly. The tensile and flexural properties therefore support the mechanical integrity of PP/CF20/CNT20 for fabrication and handling as an injection-molded electrode. However, because the mechanical properties were not re-measured after electrolysis, these results do not establish long-term electrochemical durability or retention of mechanical properties after operation.

### 3.3. Microstructural Characteristics of the Selected PP/CF20/CNT20 Composite

Figure 3a shows CFs distributed throughout the observed fracture surface of the injection-molded PP/CF20/CNT20 composite without pronounced large-scale fiber bundling. Figure 3b shows the cylindrical morphology of a representative CF, whereas Figure 3c shows torn and folded material surrounding the fibers, interpreted as a PP-rich matrix region. Figure 3d shows much finer web-like fibrillar features near the PP/CF interface.

The coexistence of distributed CFs, PP-rich matrix regions, and fine interfacial fibrillar features is consistent with a multiscale morphology in the molded composite. The selected PP/CF20/CNT20 formulation exhibited a mean volume resistivity of 0.1409 Ω·cm, corresponding to an equivalent conductivity of approximately 7.10 S cm^−1^. Within the screening set, increasing the CNT content from 10 to 20 wt.% at a fixed CF content of 20 wt.% reduced the mean volume resistivity from 0.4587 Ω·cm for PP/CF20/CNT10 to 0.1409 Ω·cm for PP/CF20/CNT20, a 69.3% decrease. However, because a composition-matched PP/CF-only control was not included and the formulation set was not factorial, the present comparison does not isolate a specific CF–CNT interaction or establish quantitative synergism.

Similar CNT-assisted conductivity enhancement has been reported in carbon-filled PP systems. King et al. [5] reported an increase in electrical conductivity from 0.29 to 17.9 S cm^−1^ after adding 6 wt.% CNT to a PP/graphite composite. Krause et al. [6] showed that electrical resistivity varied with filler combination at a fixed total filler volume fraction, while Knapp et al. [10] demonstrated improved through-plane conduction by adding CB to extruded PP/CNT films. These studies provide context for the resistivity decrease observed at the higher CNT loading; however, the present SEM images do not directly establish conductive-network connectivity or the spatial distribution of CNTs.

**Figure 4:**
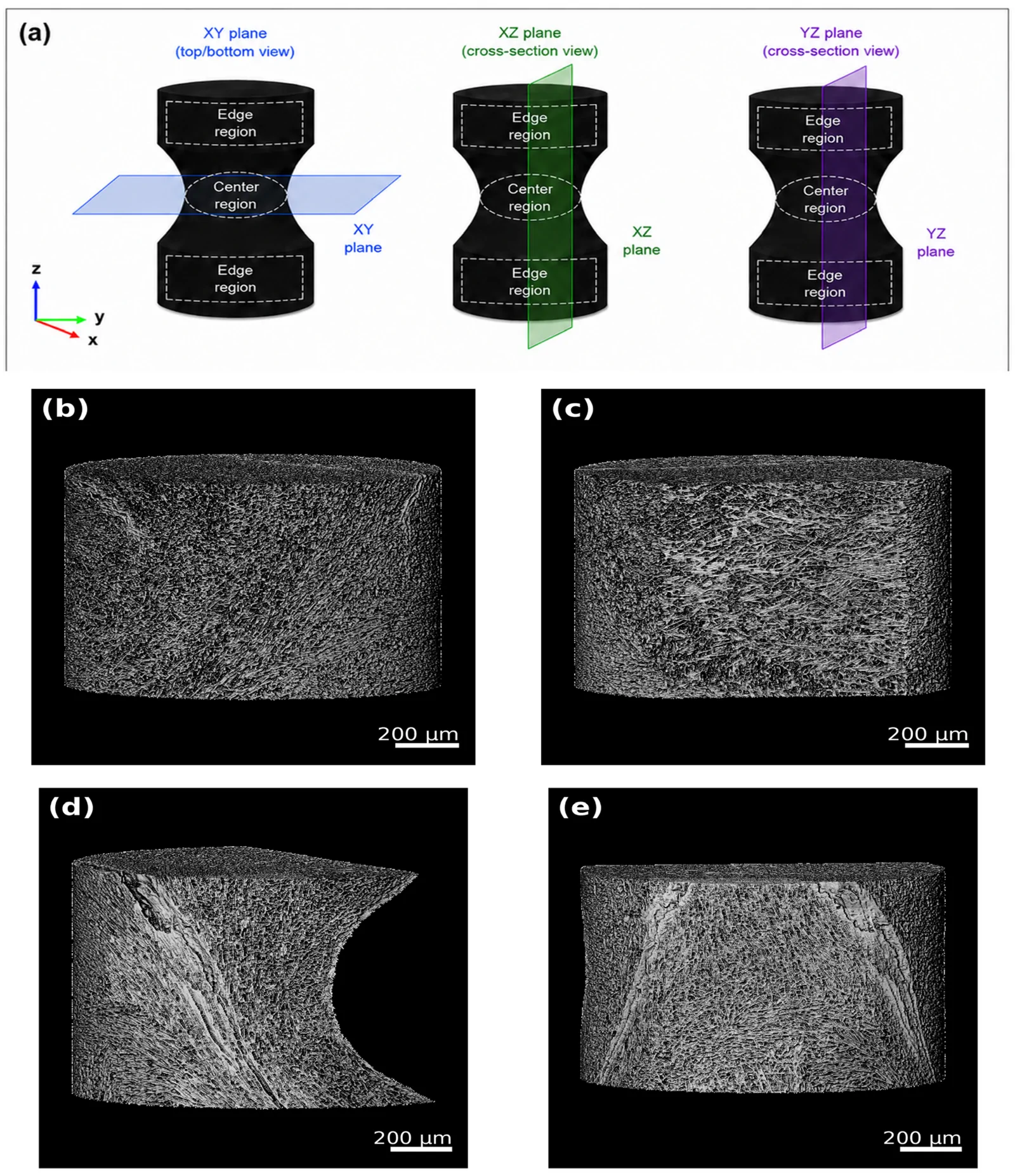
Synchrotron X-ray CT assessment of the web region between perforations in injection-molded PP/CF20/CNT20: (**a**) observation geometry and sectioning planes; (**b**,**c**) center region; (**d**,**e**) edge region. Scale bars = 200 µm.

Synchrotron X-ray CT provided a complementary, non-destructive view of CF-scale organization over volumes substantially larger than the local SEM fields. Figure 4a schematically defines the center and edge observation regions together with the XY, XZ, and YZ planes. Figure 4b,c show representative three-dimensional renderings of the center region, whereas Figure 4d,e show the corresponding edge-region views. CF-scale features were distributed throughout both reconstructed regions. The center views showed a broadly multidirectional arrangement, whereas the edge views exhibited more locally organized and curved CF-scale features near the specimen boundary. Because the analysis was based on visual inspection of reconstructed volumes rather than on segmentation-based orientation measurements, these differences are interpreted qualitatively.

X-ray microtomography studies reported in the literature have shown that fiber orientation in injection-molded short-fiber thermoplastics varies with spatial position and local flow conditions. Dupuis et al. quantified through-thickness fiber orientation in injection-molded PP/hemp composites and reported a characteristic shell–core structure, whereas Żurawik et al. used µCT-derived orientation profiles to distinguish shell and core regions and showed that local flow conditions can alter the orientation pattern [28,29]. Hessman et al. likewise demonstrated spatial variations in fiber orientation and fiber volume fraction produced by injection molding and analyzed these features using individual-fiber segmentation and orientation distributions [30]. In contrast to these quantitative approaches, the present CT analysis is intentionally conservative: the visible center–edge contrast is consistent with processing-induced microstructural heterogeneity but does not establish a quantified skin/shell/core orientation profile. SEM identifies local CF distribution and interfacial morphology, whereas CT shows CF-scale organization over larger specimen volumes. These observations provide structural context for the measured electrical and mechanical properties of the injection-molded composite, while the electrolysis tests confirm its functionality as an electrode.

### 3.4. Electrode Fabrication and Electrolysis Performance

The electrochemical functionality of the injection-molded PP/CF20/CNT20 electrodes was evaluated using the parallel-connected stack under aqueous electrolysis conditions. The experiments were designed as a proof-of-concept demonstration that the highly filled conductive PP composite could sustain electrochemical current and gas evolution. Accordingly, the present system was evaluated to demonstrate electrode functionality rather than to benchmark hydrogen-production efficiency against conventional electrolyzers.

**Figure 5:**
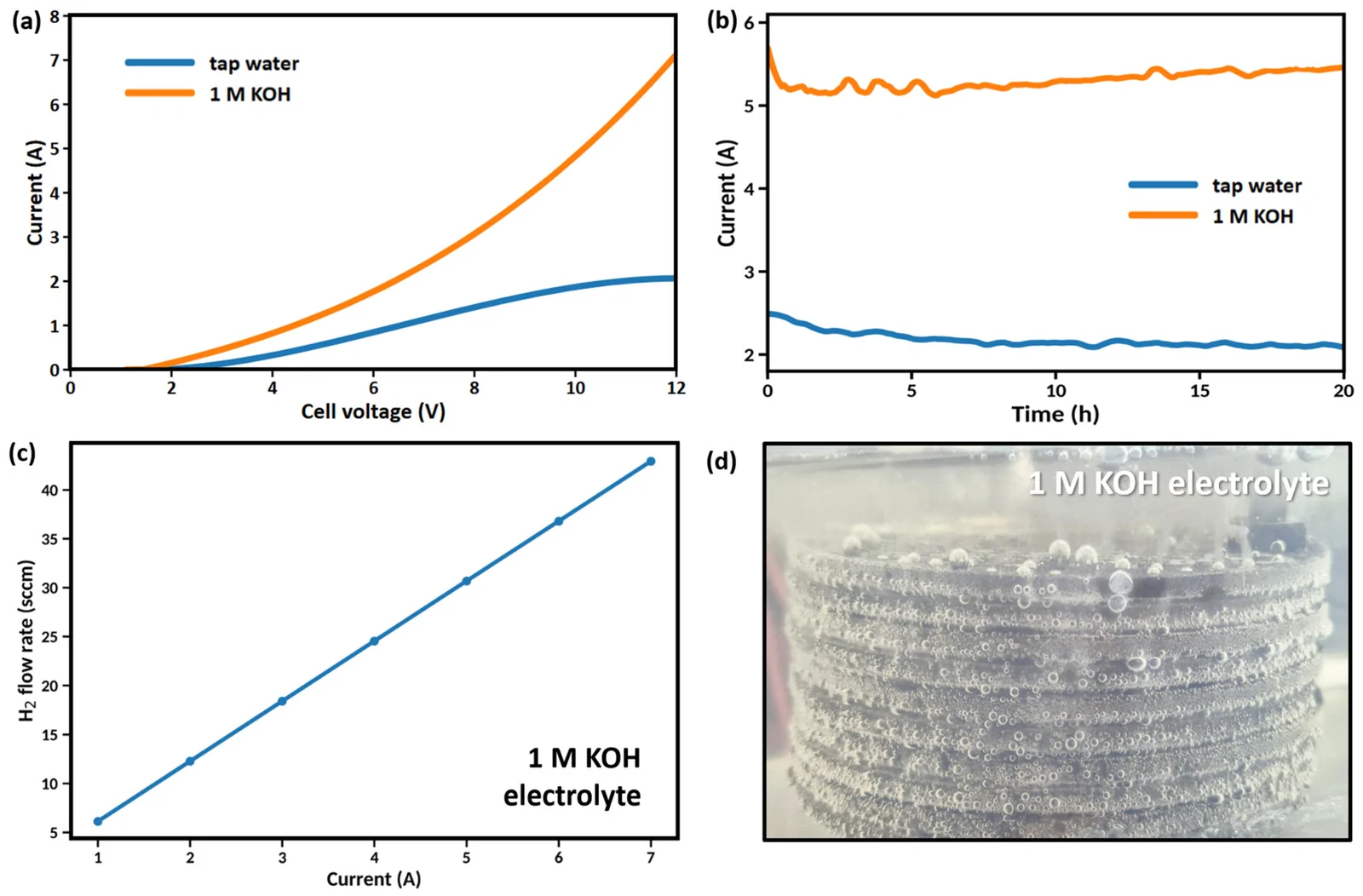
Electrolysis performance of the parallel-connected PP/CF20/CNT20 electrode stack: (**a**) current–voltage characteristics in tap water and 1 M KOH; (**b**) current response during continuous operation at 12 V for 20 h; (**c**) estimated H_2_ flow rate as a function of current in 1 M KOH, calculated from the measured mixed H_2_/O_2_ gas flow assuming a 2:1 volume ratio; (**d**) representative gas-bubble formation during operation in 1 M KOH.

Figure 5a compares the current–voltage responses of the parallel-connected stacked electrode system in tap water and 1 M KOH. The current increased with increasing applied voltage in both electrolytes, confirming that the molded composite electrodes were capable of sustaining electrical current under immersed electrolysis conditions. The response was substantially enhanced in 1 M KOH, reaching approximately 7.1 A at 12 V compared with approximately 2.1 A in tap water. This difference is consistent with the greater ionic conductivity of the KOH electrolyte, which reduces electrolyte-related ohmic resistance and facilitates ionic transport between oppositely polarized electrodes. The current measured at the end of the voltage sweep was higher than the subsequent steady-state current under prolonged 12 V operation, consistent with the initial current decay observed in Figure 5b. The observed current response is consistent with the low bulk resistivity and conductive filler morphology of the PP/CF20/CNT20 composite, although the present measurements do not independently resolve the contribution of individual conductive fillers.

The perforated geometry also provided a comparatively large electrolyte-accessible surface area. Based on the electrode dimensions, the two exposed planar faces, the internal surfaces of 220 through-holes with a diameter of 2 mm, and the outer circumferential edge, while excluding the areas covered by the electrical-connection hardware, the estimated wetted geometric surface area was approximately 182.8 cm^2^ per electrode. Notably, the internal walls of the through-holes alone contributed approximately 41.5 cm^2^, corresponding to about 22.7% of the estimated total wetted area. For five electrodes of a given polarity, the total wetted geometric area was therefore approximately 914 cm^2^. When the total stack current was normalized by this area, the apparent wetted-area-normalized current densities at 12 V were approximately 7.8 mA cm^−2^ in 1 M KOH and 2.3 mA cm^−2^ in tap water. These values are used here to describe the response of the present electrode geometry and should not be interpreted as electrochemically active surface area (ECSA)-normalized catalytic current densities. The perforations increase the electrolyte-accessible geometric area and may also provide pathways for electrolyte penetration and gas release within the stacked assembly.

The time-dependent current response at a fixed applied voltage of 12 V is shown in Figure 5b. In 1 M KOH, the current decreased initially and subsequently remained at approximately 5.2–5.5 A over the 20 h test period. In tap water, a larger initial decrease was observed, followed by stabilization near approximately 2.1 A. Although the initial transient behavior may reflect changes in electrode wetting, gas-bubble coverage, interfacial conditions, or thermal equilibration, these contributions cannot be distinguished from the present measurements alone. More importantly for the objective of this study, the molded PP/CF20/CNT20 electrodes remained electrically functional throughout continuous operation, demonstrating that the highly filled conductive PP composite was not limited to short-duration current conduction but could sustain an aqueous electrolysis response over an extended period.

Gas evolution provided additional evidence that the measured electrical response was accompanied by water-electrolysis reactions. Because the gases generated at the anodic and cathodic electrodes were collected together without separation, the gas flow measured during electrolysis represented a mixed H_2_/O_2_ stream. The mixed-gas flow rate was directly measured at each applied current in 1 M KOH, and the corresponding H_2_ flow rate was estimated by assuming that the collected gas consisted predominantly of H_2_ and O_2_ at the theoretical stoichiometric volume ratio of H_2_:O_2_ = 2:1. Accordingly, the H_2_ contribution was calculated as two-thirds of the measured mixed-gas flow rate. As shown in Figure 5c, the estimated H_2_ flow rate increased nearly linearly with increasing current, from approximately 6.1 sccm at 1 A to approximately 42.9 sccm at 7 A. This systematic increase in the estimated H_2_ flow rate with current indicates that the electrical current sustained by the highly filled PP/CF20/CNT20 composite was accompanied by progressively enhanced gas-evolving electrochemical reactions. Because the H_2_ values were derived from directly measured mixed-gas flow rather than from independently separated hydrogen, they should be interpreted as estimated H_2_ flow rates. In addition, because the flow meter was nominally calibrated for H_2_ while an H_2_/O_2_ mixed-gas stream was introduced, the converted H_2_ flow rates should be regarded as semi-quantitative estimates rather than absolute measurements of hydrogen-production efficiency.

Extensive bubble formation was visually observed over the electrode surfaces, along the perforations, and within the interelectrode spaces during operation in 1 M KOH (Figure 5d). This observation is qualitatively consistent with the higher current response in the alkaline electrolyte and with the increase in estimated H_2_ flow derived from the directly measured mixed-gas flow shown in Figure 5c. Taken together, the I–V response, 20 h current behavior, current-dependent mixed-gas generation, and visible bubble evolution demonstrate that the injection-molded PP/CF20/CNT20 composite can function as a self-supporting electrode for aqueous electrolysis while containing 40 wt.% conductive carbon fillers. Accordingly, the present electrolysis tests should primarily be interpreted as a proof-of-concept validation of the electrochemical functionality of an injection-moldable conductive thermoplastic electrode rather than as an optimization study for commercial hydrogen production. Further optimization of electrode composition, cell architecture, operating voltage, gas-separation configuration, gas-flow calibration, and long-term operating conditions would be required before evaluating the system in terms of practical hydrogen-production efficiency.

**Figure 6:**
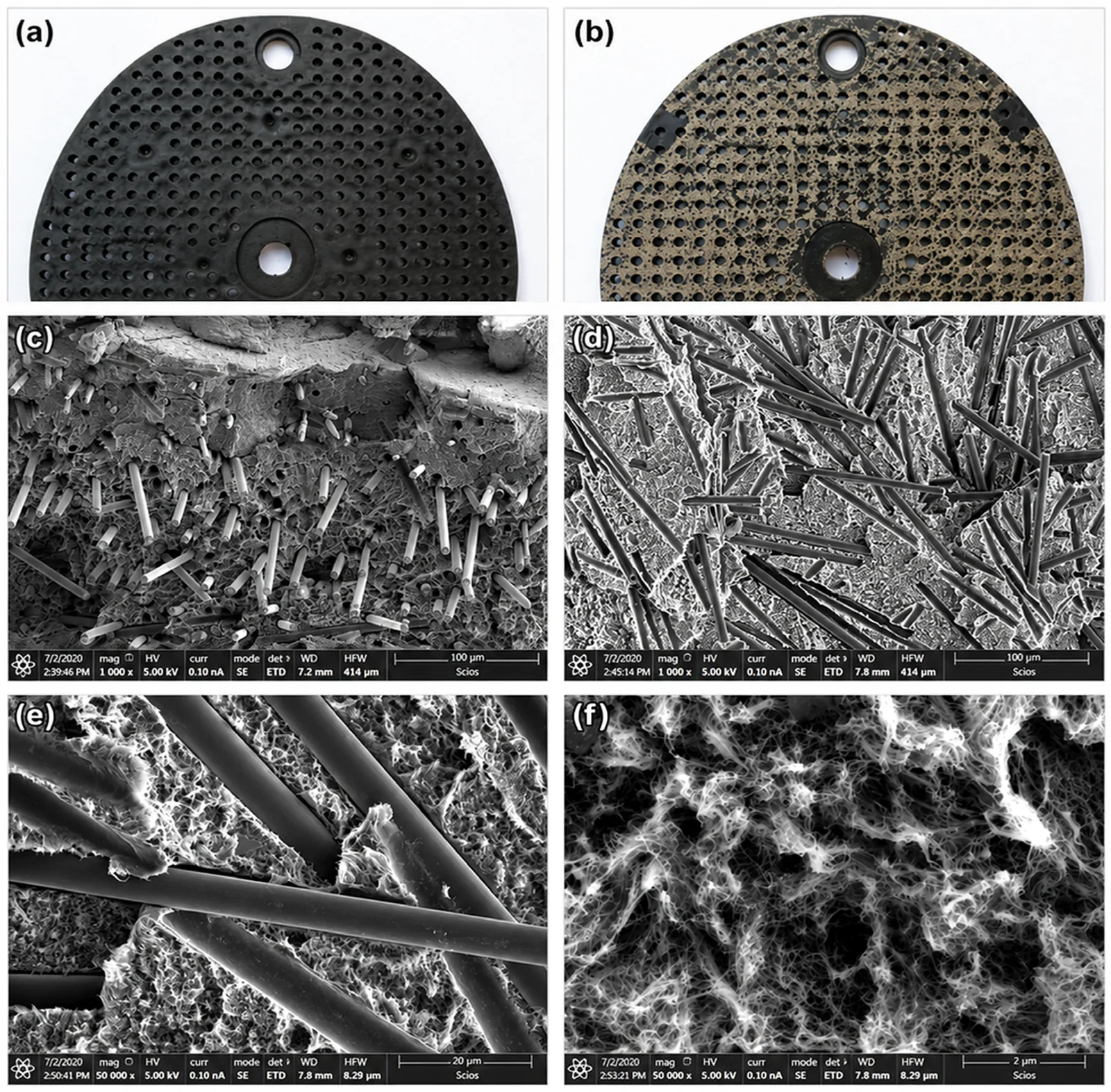
Macroscopic and SEM observations of PP/CF20/CNT20 electrodes before and after 120 h of electrolysis in tap water: (**a**) unused electrode; (**b**) used electrode with cathodic scales; (**c**–**f**) SEM images of post-electrolysis fracture surfaces.

Figure 6a,b compare the macroscopic appearance of unused PP/CF20/CNT20 electrodes and those continuously operated for electrolysis in tap water for 120 h, respectively. The unused electrode exhibited a predominantly uniform black surface, whereas the used electrode showed extensive light-colored deposits over the exposed surface and around the perforations, indicating substantial scale formation during operation. Because the fracture-surface morphology of the unused composite is shown in Figure 3, Figure 6c–f focus on the post-electrolysis internal morphology.

Figure 6c,d show that CF-scale features remained readily identifiable throughout the examined fracture surfaces and were embedded within the surrounding PP-rich matrix. No obvious large-area disruption or filler-depleted regions were observed in these representative fields.

Figure 6e shows individual CF surfaces. Figure 6f shows fine fibrillar features in the matrix region near the PP/CF interface after electrolysis, morphologically similar to those observed in Figure 3d prior to electrolysis. Despite substantial external scale formation, no obvious large-scale alteration of the internal composite morphology was evident in the examined regions. Because the unused and used specimens were obtained from different physical locations, this comparison is qualitative and does not establish filler retention or long-term structural stability.

**Figure 7:**
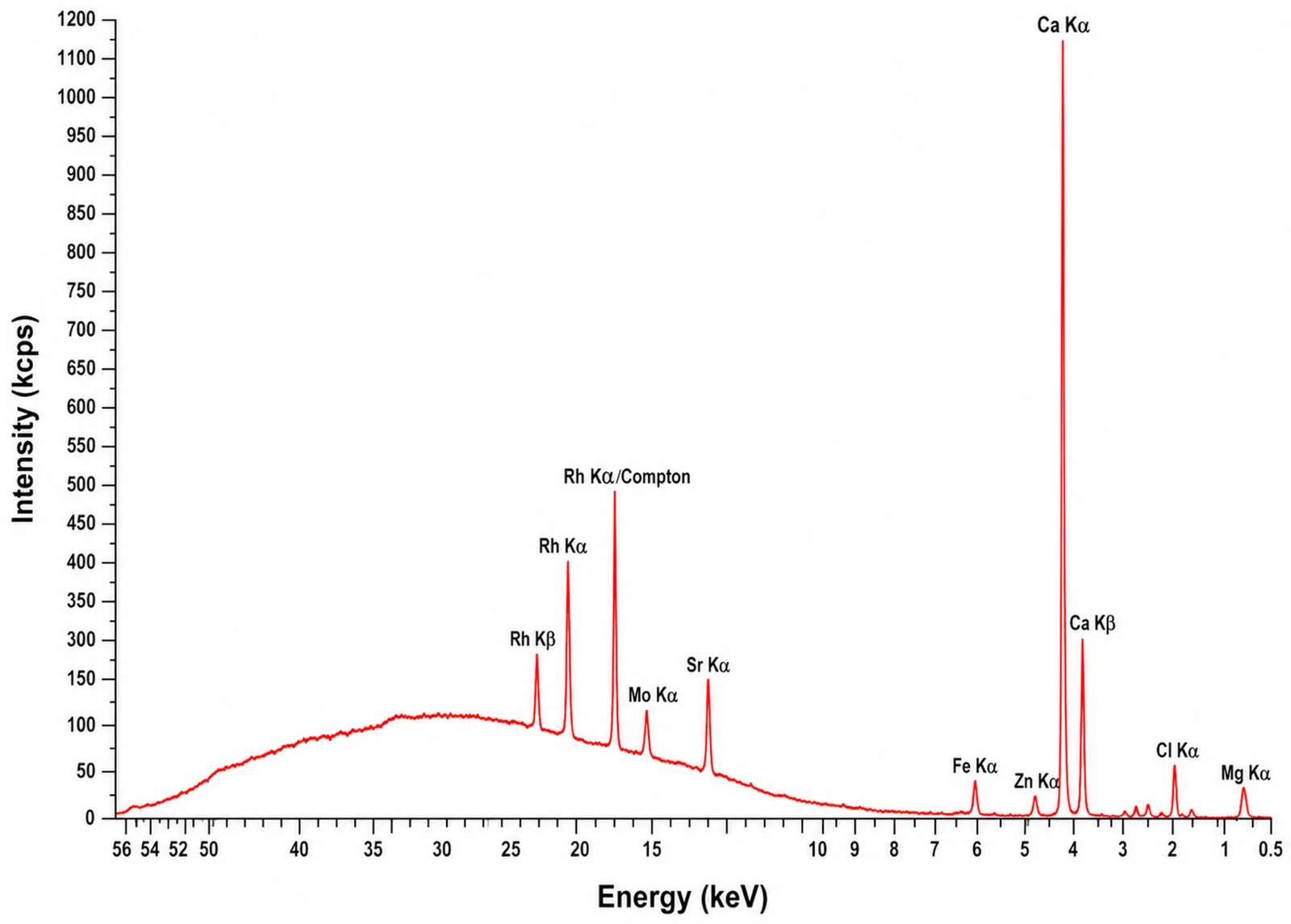
XRF spectrum of cathodic scale sample collected after 120 h of tap-water electrolysis.

XRF analysis of the cathodic scale sample showed that the deposit was dominated by CaO-equivalent content (95.76 wt.%), with substantially smaller contributions from Cl (2.16 wt.%) and MgO (0.723 wt.%) and only trace levels of the remaining detected components (Figure 7). The deposit was therefore Ca-dominated, with minor Cl- and Mg-bearing constituents.

Cathodic generation of OH^−^ increases the local interfacial pH and can promote precipitation of hardness minerals such as CaCO_3_ and Mg(OH)_2_ in Ca^2+^/Mg^2+^-containing water [17,18]. The Ca-dominated XRF composition is consistent with cathodic calcareous-scale formation; however, XRF alone cannot distinguish CaO, CaCO_3_, Ca(OH)_2_, or other Ca-bearing phases and does not reliably detect carbon or hydrogen under the present measurement conditions. Definitive phase identification would therefore require X-ray diffraction or a complementary vibrational-spectroscopic method.

Ca^2+^ + HCO_3_^−^ + OH^−^ → CaCO_3_(s) + H_2_O Mg^2+^ + 2OH^−^ → Mg(OH)_2_(s)

### 3.5. Electrochemical Inactivation of Escherichia coli

**Figure 8:**
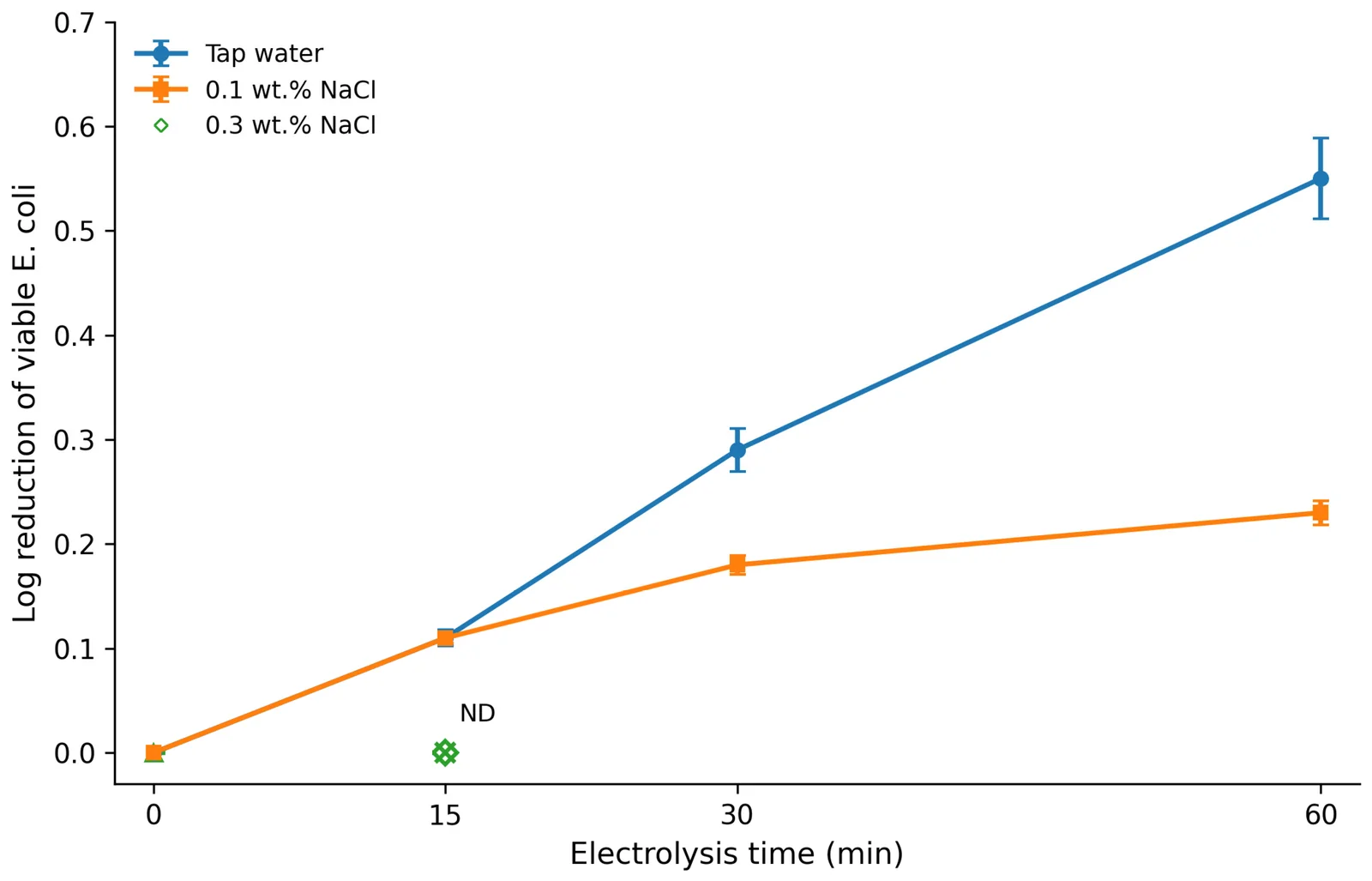
Mean log_10_ reduction in culturable *Escherichia coli* during electrolysis at 12 V in tap water and 0.1 wt.% NaCl (error bars indicate SD). For 0.3 wt.% NaCl, the crossed open-diamond marker at 15 min indicates that no colonies were detected from 15 min onward; it does not represent a numerical log-reduction value.

Following operational verification of the molded composite electrodes, the electrode system was evaluated for *E. coli* inactivation at a fixed cell voltage of 12 V. Figure 8 presents mean log-reduction values from three independent experiments for the quantifiable tap-water and 0.1 wt.% NaCl data, with error bars indicating SD. For the 0.3 wt.% NaCl condition, no colonies were detected in any of the three independent experiments from 15 min onward. Because the quantitative detection limit could not be reconstructed, these observations are reported as ND and were not assigned numerical log-reduction values.

In tap water, the mean log reduction increased progressively to 0.55 at 60 min, corresponding to a 71.5% reduction relative to the initial viable count. The tap water used in the experiments was potable municipal water. Available water-quality information indicated a residual chlorine concentration of approximately 0.2 mg/L (0.2 ppm). This residual disinfectant, together with other dissolved ionic species, may have contributed to the observed response; however, its contribution cannot be quantified because residual chlorine was not measured concurrently during the *E. coli* treatment. In the 0.1 wt.% NaCl condition, the mean log reductions were 0.11, 0.18, and 0.23 at 15, 30, and 60 min, respectively, indicating a less pronounced response than that observed in tap water over the same treatment period.

By contrast, the 0.3 wt.% NaCl condition showed a markedly more rapid loss of culturability. The measured bulk-water temperature remained within 19.6–20.9 °C during this test, suggesting that bulk-water heating was not the dominant treatment factor. Overall, the results indicate that the inactivation response depended on the water matrix and electrolyte composition. The rapid response under the 0.3 wt.% NaCl condition is qualitatively consistent with chloride-assisted electrochemical inactivation. However, differences in test volume and the absence of condition-specific current and cumulative-charge measurements preclude attribution of the response solely to NaCl concentration. Because a time-matched no-voltage control was not included, the contribution of electrolysis could not be fully separated from background changes in bacterial culturability or matrix-specific disinfectant effects.

The rapid inactivation observed at 0.3 wt.% NaCl is qualitatively consistent with chloride-assisted electrochemical disinfection. In chloride-containing electrolytes, anodic oxidation of Cl^−^ can generate Cl_2_, which subsequently forms HOCl/OCl^−^ in water. Previous electrochlorination studies have shown that chloride concentration, current density, and water composition can influence reactive-chlorine generation and bacterial inactivation [16,20,21,22,23,25]. Ghasemian et al. reported increased bactericidal activity with increasing NaCl concentration and identified reactive chlorine species as important contributors under their experimental conditions [25]. Long et al. further showed that *E. coli* inactivation in a chloride electrolyte was associated predominantly with damage to intracellular enzymatic systems [24], consistent with the intracellular effects of free chlorine described by Cho et al. [23]. These literature mechanisms provide a plausible context for the present 0.3 wt.% NaCl response but were not directly measured in this study. Because free and total chlorine, current density, and cumulative charge were not determined concurrently with the microbiological tests, the present results support a chloride-assisted interpretation rather than direct evidence of a specific reactive-chlorine pathway.

## 4. Conclusions

This study demonstrated that a highly filled PP-based conductive composite can be melt-compounded and injection-molded into a functional electrode that combines sufficient mechanical integrity for fabrication and handling with sustained electrochemical functionality. Among fifteen formulations, PP/CF20/CNT20 was selected because it combined a low mean volume resistivity of 0.1409 Ω·cm with complete mold filling, no visible surface defects, and good overall moldability. SEM and synchrotron X-ray CT provided complementary information on the microstructural organization of the molded composite.

The ten-electrode parallel stack sustained electrolysis in tap water and 1 M KOH. At 12 V, the voltage sweep reached approximately 7.1 A in 1 M KOH and 2.1 A in tap water. During 20 h fixed-voltage operation, the currents stabilized at approximately 5.2–5.5 A and 2.1 A, respectively. In 1 M KOH, the estimated H_2_ flow rate increased from approximately 6.1 sccm at 1 A to 42.9 sccm at 7 A. Because H_2_ and O_2_ were collected as a mixed stream and H_2_ was estimated from the theoretical 2:1 volume ratio, these gas-flow values should be regarded as semi-quantitative. After 120 h of tap-water electrolysis, extensive surface scaling was observed, and XRF showed a Ca-dominated deposit. Under the same 12 V operating voltage, the electrode system also reduced viable *Escherichia coli*, with no colonies detected under the applied plating conditions from 15 min onward in 0.3 wt.% NaCl.

The present study was not intended to establish a performance comparison between the composite electrode and conventional metal-based electrolysis electrodes. Rather, its potential advantages may include manufacturability and replaceability. The manufacturing advantages of injection molding may facilitate standardized production and replacement of scale-fouled electrodes. However, the economic viability of such a replacement strategy remains to be established. Future work should therefore evaluate manufacturing cost, long-term electrical and mechanical stability, scale-related performance degradation, gas-separation and Faradaic efficiencies, controlled disinfection performance with simultaneous current/charge and reactive-chlorine measurements, and practical replacement intervals under representative water chemistries.

## Figures and Tables

**Table 1:** Representative electrical properties of melt-processed thermoplastic/carbon-filler composites reported in the literature.

Study	Polymer Composite	Processing/Test Specimen	Reported Conductivity	Equivalent Resistivity
Pathak et al. (2021) [11]	PP/CNF, 60 wt.% CNF	Melt extrusion; injection-molded specimen	1.4 S cm^−1^	0.71 Ω·cm *
Pathak et al. (2021) [11]	PP/GRP, 60 wt.% GRP	Melt extrusion; injection-molded specimen	0.4 S cm^−1^	2.50 Ω·cm *
Chen et al. (2022) [2]	PP/CF, 30 wt.% CF	Twin-screw compounding; MuCell/GCP injection-molded specimen; through-plane	1.7865 S m^−1^	56.0 Ω·cm *
Paleo et al. (2022) [12]	PP/CNF, 1.4 vol.% (~3 wt.%)	Melt extrusion; compression-molded 10 mm × 10 mm × 0.5 mm specimen; DC	8 × 10^−6^ S m^−1^	1.25 × 10^7^ Ω·cm *
Tariq et al. (2023) [1]	PP25/Gr36/MWCNT4/EG30/CB5 (wt.%)	Twin-screw extrusion and pelletization; compression-molded 1.5 mm disc; through-plane	39.6 ± 1.1 S cm^−1^	0.0253 Ω·cm *
Knapp et al. (2026) [10]	PP/MWCNT, 2 wt.% MWCNT	Continuously extruded film; X, in-plane; Z, through-plane	Not reported	0.9 ± 0.1/73.5 ± 10.0 Ω·cm

* Calculated as ρ = 1/σ after unit conversion. Values are not directly comparable because specimen geometry, processing conditions, measurement direction, and contact methods differed among studies. Abbreviations: CNF, carbon nanofiber; GRP, graphite; MWCNT, multiwalled carbon nanotube; EG, expanded graphite; CB, carbon black.

**Table 2:** Mean volume resistivity and qualitative injection-molding outcomes of the screened PP/carbon-filler formulations.

Nominal Formulation (wt.%)	Mean Volume Resistivity (Ω·cm)	Mold Filling	Surface Defect	Overall Moldability
PP/CF30/Gr30	1.3239 ± 0.170	Difficult	Observed	Poor
PP/CNT5/Gr40	2.2730 ± 0.340	Difficult	Minor	Moderate–Poor
PP/CF20/CNT5/Gr20	0.1658 ± 0.015	Difficult	Minor	Moderate
PP/CNT10/Gr20	0.2262 ± 0.023	Complete	Minor	Good–Moderate
PP/CF15/CNT10/Gr15	0.4751 ± 0.048	Complete	Minor	Good–Moderate
PP/CF15/CNT10/Gr25	0.2125 ± 0.013	Difficult	Minor	Moderate
PP/CF20/CNT10	0.4587 ± 0.046	Complete	None	Good
PP/CF20/CNT10/Gr20	0.2392 ± 0.014	Difficult	Minor	Moderate
PP/CF25/CNT10/Gr15	0.1329 ± 0.010	Difficult	Observed	Moderate–Poor
PP/CF30/CNT10/Gr10	0.1261 ± 0.010	Difficult	Observed	Moderate–Poor
PP/CF20/CNT20	0.1409 ± 0.011	Complete	None	Good
PP/CNT20/Gr20	0.1529 ± 0.014	Difficult	Observed	Moderate–Poor
PP/CF20/CNT10/CB10	0.2284 ± 0.023	Difficult	Minor	Moderate
PP/CF15/CNT10/CB10	0.2022 ± 0.020	Complete	Minor	Good–Moderate
PP/CF15/CNT10/CB15	0.1640 ± 0.013	Difficult	Minor	Moderate

Values are mean ± SD of five specimen-level values (*n* = 5 specimens).

**Table 3:** Representative properties of the selected injection-molded PP/CF20/CNT20 composite.

Property	Test Standard/Method	Value	Unit
Volume resistivity	Four-point probe	0.1409	Ω·cm
Tensile strength	ASTM D638	100.9	MPa
Tensile elongation	ASTM D638	1	%
Flexural strength	ASTM D790	168.5	MPa
Flexural modulus	ASTM D790	18.87	GPa

## Data Availability

The original contributions presented in this study are included in the article. Further inquiries can be directed to the corresponding authors.
